# Back Pain Among Paramedics: A Pilot Study

**DOI:** 10.5812/nms.12195

**Published:** 2013-12-10

**Authors:** Mohsen Adib-Hajbaghery, Javad Zohrehea

**Affiliations:** 1 Trauma Nursing Research Center, Kashan University of Medical Sciences, Kashan, IR Iran

**Keywords:** Back Pain, Allied Health Personnel, Musculoskeletal Diseases

*Dear Editor,*

The back pain is one of the most prevalent health problems, and about 60-80% of people encounter with the same problem at least once in their whole life time (1). Several studies have shown that some healthcare workers are at high risk of musculoskeletal disorders, particularly low back pain (2). The Nurses and paramedic staffs, especially ambulance paramedics who work in prehospital emergency medical system (PEMS), are at high risk of back pain due to the nature of their services, encountering stressful conditions, strenuous physical activities such as handling patients, bad work environment, and long-time standing (3-5). Additionally, ambulance paramedics are vulnerable to physical disorders that cause back pain not only because they involve in caring, handling injured patients, and transporting patients with acute or chronic disorders, but also should work under abnormal circumstance such as the scene of accidents, natural disasters, and the confined space in the ambulance. However, few studies are available on the prevalence of low back pain and musculoskeletal disorders among ambulance paramedics, but no study on this important issue is available from Iran. Therefore, this study aimed to investigate the rate of low back pain and the contributory factors among ambulance paramedics who worked in the PEMS of Kashan, a city in the central region of Iran, in May 2012. A descriptive study was conducted on all ambulance paramedics in the PEMS of Kashan. A questionnaire was used for data collection. This questionnaire consisted of questions regarding demographic features, and levels of psychological, social and job related stress as well as the stress related to the work environment. Content validity of the questionnaire was confirmed by five faculty members in the Faculty of Nursing and two officials of the PEMS. Also the reliability of the questionnaire confirmed using test-retest on five paramedics (r = 0.73 to 0.81 for different parts). A total of 68 questionnaires were distributed among paramedics and 55 questionnaires were returned. Seven questionnaires were excluded as they were responded incompletely, finally 48 questionnaires were analyzed. To analyze the data, descriptive statistics, chi-square tests and student’s t-tests were used. Half of the participants were graduated nurses, working in PEMS and the others were technicians in emergency medicine. In total, 79.17% of the participants had experienced at least one episode of low back pain in the last six months from the time of data collection. Although nearly 90% of participants expressed that they know the correct manner of transporting a patient with a stretcher and a long backboard, 47.37% of the back pains have been occurred after lifting and transporting heavy objects or patients. Significant relationships were observed between low back pain and work experience (P = 0.025), body mass index (P = 0.001), job dissatisfaction (P = 0.04), level of work-related psychological stressors, and fatigue due to overwork (P = 0.01). The low back pain was not related to the levels of impatience and depressed mood (P = 0.256), levels of qualification (P = 0.872), and anxiety in the workplace (P = 0.7). The results of this study showed that low back pain is a serious problem which is prevalent among paramedics of the PEMS. This may affect their efficiency and quality of life. Lifting heavy patients was the most common cause of back pain. Also the study showed that the mental condition of the participants plays a significant role in their low back pain. Therefore, the authorities should pay attention to the education of the paramedics about the ergonomics. Moreover establishing workplace assessment program, rehabilitation, refreshing, and stress reducing strategies should be considered. 
